# How do patients access and experience long-term care after stroke in the German healthcare system? A qualitative interview study

**DOI:** 10.1136/bmjopen-2024-090206

**Published:** 2025-06-30

**Authors:** Sofia Banzhoff, Christoph Heintze, Susanne Döpfmer, Christine Holmberg

**Affiliations:** 1 Institute of General Practice and Family Medicine, Charité – Universitätsmedizin Berlin, corporate member of Freie Universität Berlin and Humboldt-Universität zu Berlin, Berlin, Germany; 2 Institute of Social Medicine and Epidemiology, Brandenburg Medical School Theodor Fontane, Brandenburg/Havel, Germany; 3 Faculty of Health Sciences, Brandenburg Medical School Theodor Fontane, Potsdam, Germany

**Keywords:** Person-Centered Care, Patient Care Management, QUALITATIVE RESEARCH, STROKE MEDICINE, Health Services

## Abstract

**Abstract:**

**Objective:**

Understanding how patients access and experience long-term care after stroke, including the kind of medical support desired, in a qualitative interview study; analysis with a question-focused approach adapted from grounded theory methodology.

**Setting:**

Recruitment in primary care and physical therapy practices in the metropolitan area of Berlin, Germany.

**Participants:**

15 patients treated in general practice or physical therapy, whose last stroke occurred more than 2 years ago.

**Results:**

‘Shaping relationships’ was the core category extracted from the data as a necessary component to receive appropriate long-term care after stroke. Care is embedded in relationships that must be managed primarily by the study participants and their proxies in the German setting. Study participants used different strategies to shape care relationships. This process is helped or hindered by healthcare institutions. Compared with concepts of patient-centred care, patients play a more active role in shaping relationships. To improve long-term care for chronic diseases, this needs to be taken into account.

**Conclusion:**

Shaping relationships is a composite skill that includes engaging in and sustaining relationships, finding and using information and communication. This skill is essential for adequate long-term care after stroke.

**Strengths and limitations of this study**

- Use of an open, qualitative approach to understand patients’ perspectives.
- A broad range of time since last stroke (2–20 years).
- Consistent data collection and intersubjective validation through interpretation groups.
- Selection of interviewees through their healthcare professionals.
- Participation was tied to being verbally competent to do an interview.

## Introduction

 The prevalence and incidence of stroke remain high despite prevention efforts. In 2019, the incidence was estimated to be 12.2 million strokes worldwide and prevalence was assumed to consist of 101 million persons worldwide. If these trends continue, the estimated global prevalence rate for stroke by 2050 is 200 million persons.^1^ In Germany, the 12-month prevalence of stroke or chronic health issues due to stroke is 2.1% in women and 2.3% in men of all ages, and as high as 5.5% in women 80 years and older and 6.2% in men aged 65–79.^2^

Furthermore, the risk of stroke recurrence is 16.1% 2 years after the initial stroke, 14.8% after 5 years and 39.7% after 12 years.^3^ Risk factors for stroke recurrence include arterial thrombosis, hypertension, hypercholesterolaemia, arterial stenoses, diabetes, sleep apnoea, a lack of physical activity, inadequate diet, smoking and high salt consumption.^4 5^ These can be modified through adequate care, such as medication, physical therapy and support in enacting lifestyle modifications. Care and rehabilitation can also improve the quality of life (QoL).^6^ Long-term care after stroke for secondary prevention^7^ is, therefore, a significant public health challenge.

Most patients who had an acute stroke in Germany are treated by neurologists in specialised stroke units in hospitals and many patients attend stroke-specific in- or outpatient rehabilitation after that.^8^ There are no structured aftercare programmes following rehabilitation; general practicioners (GPs) tend to be the main healthcare professional (HCP) for follow-up care. Problems can arise as the German healthcare system has a more rigid separation into sectors than other countries. Physicians working in hospitals only see inpatients, whereas follow-up care takes place in ambulatory practices of GPs or other specialists. This can lead to logistical issues and gaps in care.^8^ Social network size was found to be positively associated with QoL in the first year after discharge from rehabilitation.^9^ Insurance is a lower barrier than in other countries as more than 90% of the population are insured via statutory health insurance and most others are insured privately.^10^

Despite the high burden of disease and high risk of recurrence, there is a dearth of research on long-term care after stroke.^11^ Currently, many patients who had a stroke in Germany receive inpatient rehabilitation, but long-term care remains a challenge as it is less structured than inpatient rehabilitation and can look very different for different patients despite having similar care needs.^12^

Common problems include limited access to outpatient care and prescriptions for physical therapy, lack of multidisciplinary coordination and support for caregivers and poor organisation of the transition between inpatient and outpatient care. These problems have an impact on health-related QoL. In a study from 2018 comparing patients’ current care to guideline-suggested care in Germany, stroke specialists found notable discrepancies.^13^ They recommended outpatient therapy (physical/speech therapy) for almost half of participants, with additional recommendations for social work intervention (33%), medical review (30%) and optimised pharmacotherapy (18%).^13^ Early rehabilitation after stroke is crucial, but so is sustaining and improving on those gains.

Strokes present challenges to healthcare systems, specifically the high burden of disease, the rising prevalence with advancing age and the high probability of strokes affecting multimorbid patients or rendering those with other pre-existing conditions multimorbid. The varied presentations of stroke sequelae—from mobility issues along a spectrum of balance problems to a complete inability to move, speech impairments, dysphagia, psychological symptoms, cognitive impairment, etc.—highlight the importance of multimodal and individualised long-term care. Studying patients who had a stroke and their long-term care can, thus, improve care systems for other chronic conditions and multimorbid patients.

The patient–provider relationship is central for long-term care. Reviews of interpersonal interventions between patients and providers have found that improving this relationship can lead to enhanced health outcomes, such as better physical functioning, obesity control, improved mental health and lower healthcare costs, among other things.^14 15^ In a study conducted in a neurology–neurosurgery outpatient clinic in Greece with patients at least 6 months after a stroke, the quality of this relationship was found to be related to compliance in taking medications 6 months and longer after an ischaemic stroke, as well as to the mental state of patients and the perceived necessity for medications.^16^ In another study looking at poststroke spasticity, patients were dissatisfied with the communication and amount of information they received. Additionally, patient and physician rehabilitation goals were often misaligned, with patients valuing improvements in motor skills higher than physicians.^17^

A study of aftercare for stroke (starting at 3-months poststroke) conducted in Berlin—the same city and healthcare system as in our study^13^—found important unmet needs and stroke-related health problems in 95% of the study participants (overall sample size: 57). These included spasticity, cognitive deficits and depression. Unmet needs were medical review, social work input (eg, due to caregiver burden), optimised pharmacotherapy and outpatient therapy.

To our knowledge, there are no studies that investigate long-term care several years after the stroke event. In addition, few have investigated patients’ own perspectives—a perspective that is crucial for compliance and understanding patients’ needs. The focus of this article is long-term care from study participants’ perspectives. The aim of the study is to understand the long-term care experiences of patients with a stroke event that occurred at least 2 years prior to study participation. To do so, we analysed semistructured, qualitative interviews with a focus on how patients discuss their access to and experiences of long-term care after stroke.

This study is a part of a larger patient-centred healthcare research project with the goal of reducing barriers and inequities in the care of patients with age-associated diseases.^18^ Patient centredness aims to respect and respond to a patient’s needs, values and preferences and to use them to guide clinical decisions.^19^ A patient-centred navigation system similar to case management was developed as a ‘connector’ between patient needs and the fragmented healthcare system.^19^,^21^ This is especially necessary as patients have been found to have significant unmet medical and social needs in their long-term care after stroke, such as outpatient therapy, optimisation of medication, input from a social worker regarding disability or their socioeconomic situation^13 22^ and decreasing QoL.^9^

## Methods

We conducted a qualitative interview study with persons who had a stroke at least 2 years prior to the interview, living in the German city of Berlin and its surrounding areas. Approval was granted from the ethics committee of the Charité–Universitätsmedizin Berlin (EA1/254/18). The design of this qualitative interview study aimed to capture patients’ perspectives and experiences in order to improve healthcare based on citizens’ own needs. The reporting of findings follows the Consolidated criteria for reporting qualitative research (COREQ).^23^

### Patient and public involvement

None.

### Recruitment and data collection

Data were collected through semistructured qualitative interviews conducted in-person in the spring and summer of 2019. Interview questions (see online supplemental table 1 for a summary) were developed for the following areas of inquiry: changes since the stroke, how limitations due to chronic illness influence daily life, current medical care and support and what other support participants wish for. The development was informed by a literature search, the authors’ clinical experience and theoretical considerations.

We recruited participants in GPs’ and physical therapists’ (PTs) practices in the Berlin metropolitan area. These practices were either a part of a research practice network, were additional places of employment of colleagues at the university or were personally known to the first author, who asked the GPs and PTs to take part in the study. Patients were then approached by their GPs or PTs and invited to participate. We limited recruitment from each practice to three participants to avoid over-representing the interaction and relationship with particular GPs or PTs. Inclusion criteria were being older than 18 years, last stroke at least 2 years prior to the interview, community dwelling and being willing and able to be interviewed.

The first author contacted participants via phone, confirmed their eligibility and made an interview appointment. They were then sent information by post on the study, informed consent and data security. Based on a review,^24^ one may assume that most crucial information in qualitative interview studies is gained within the first six interviews, with the rate of new information declining sharply following an asymptotic curve. Our aim was to interview 15 participants to get an overview of different long-term care situations after stroke and ensure data saturation for our research questions. Previous research by the authors SD and CHe also supports such sample sizes.^25^

The semistructured interviews were conducted in participants’ homes. They had the option of their spouse or another trusted person being present. All interviews were conducted by the first author (SB), who was a medical student at the time. This was her first independent research project, which she was conducting for her MD dissertation. Participants were aware of this and did not receive financial compensation.

Demographic questions were limited to name, date of birth, civil status and zip code for data security reasons. The interviewer took field notes after each interview, which were referred to for context when necessary.

### Transcription and data analysis

All interviews were audio recorded and transcribed verbatim in MAXQDA 2018 (analysis in MAXQDA 2018 and later 2020) by the first author. The transcripts were not returned to interview partners. During transcription, any identifying information was removed. The first author then read and reread the transcripts to familiarise herself with the data and presented the interviews in interpretation groups.

Initially, only a thematic analysis regarding unmet needs in long-term care after stroke was envisioned. However, to adhere to the principle of openness and responsiveness to the data as important quality assurance criteria of qualitative research, we complemented this with a more open-ended and question-focused analysis related to a grounded theory approach once we saw the richness of the data with regards to our research questions and the limitations a sole thematic analysis would have posed. Thus, rather than focusing on a preconceived notion of ‘unmet needs’, we investigated patients’ experiences of care. To be true to the data, the data were analysed according to the following questions: what does it mean from patients’ perspectives to access and experience long-term care after stroke? How do they experience being cared for, receiving support for this care and receiving care? Following the grounded theory approach, the aim then became to develop a core category that explains the data in relation to care. Steps in this process were open and focused coding, constant comparison and memo writing.

The first author identified all passages concerning participants’ strategies for care: descriptions of actions or interactions, participants’ evaluations of these processes, any mention of goals and care needs and wishes or questions regarding care. These passages from five maximally contrasting interviews were coded line-by-line using open coding.^26^ We chose contrasting interviews based on gender, the severity of their condition, living with a spouse versus living alone and how well their care was organised.

During focused coding,^26^ the first author developed preliminary subcategories by choosing the open code that best described a given passage. These were further developed using memos and sorted into preliminary categories. These categories and subcategories were defined and further developed by writing memos; overlapping subcategories were combined and sorting into categories was adjusted as needed. After this, about 180 subcategories in 14 categories remained. Throughout this process, the first and last author held data sessions at every step of the analysis process to discuss identified passages, coding, the coding system, memos and categorisations. They coded passages together and discussed the first author’s codes of other passages in detail. Disagreements were resolved via discussions until they reached a consensus.

In the review of categories and subcategories and detailed discussion by the first and last author, a core category emerged that encompassed all categories and subcategories and explained the unique challenges of long-term care after stroke. This was tested by writing memos linking each category and subcategory to the core category. Preliminary results were discussed with several research groups and colloquiums at our institution.

The core category was developed further by applying it to the ten remaining interviews. This helped to identify its properties and patterns. The analysis of the last five interviews did not add any new aspects to the developed concept.^26^ There was no participant checking of our findings.

## Results

We recruited 15 participants from 7 primary care and 2 physical therapy practices and conducted 15 semistructured interviews in German, the native language of the authors and all but 1 participant. The median interview length was 49 min (range 20–113). 11 interviews were one-on-one; in 4, another person was present and contributed (3 spouses and 1 friend). No participant stopped the interview or dropped out later. Participant characteristics are listed in table 1.

**Table 1:** Characteristics of participants

Age (in years)	Median 73 (Range: 57–91)
Gender	8 female, 7 male, 0 non-binary
Time since last stroke (in years)	Median 6 (Range: 2–20)
Living situation	5 alone, 10 with spouse
Relationship status	12 in relationship, 3 single

The core category that developed out of the analysis was ‘shaping relationships’. Indeed, to receive the care that our interview partners described in positive terms or considered appropriate, specific relationships were necessary. In the following, we describe these relationships and the strategies used to shape them.

**Figure 1:**
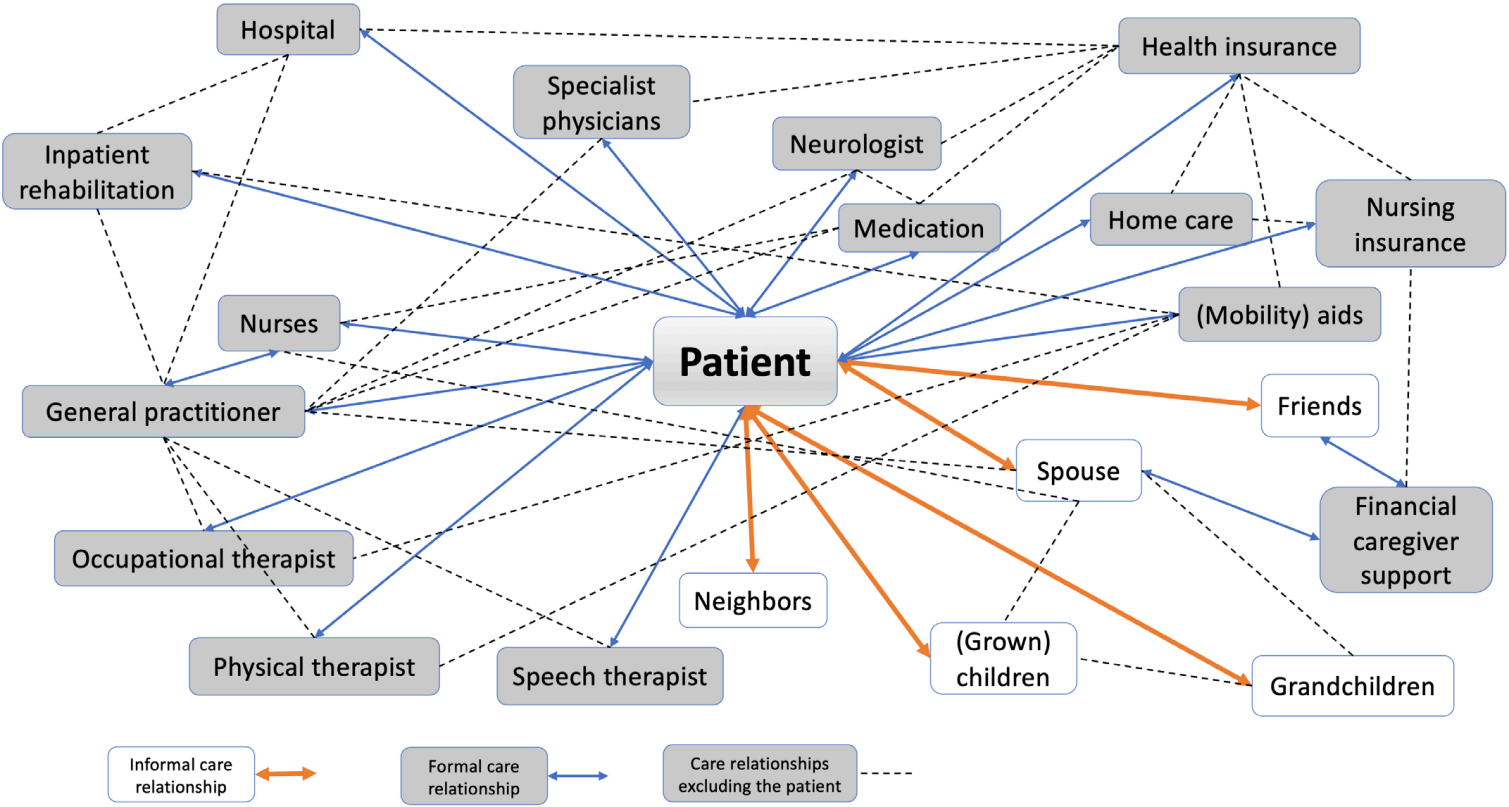
Web of formal and informal care relationships. Orange/bold arrow: informal care relationship. Blue/fine arrow: formal care relationships. Broken line: care relationships excluding the patient.

To receive appropriate care, participants need to shape the relationships within which care takes place. This involves all actions or omissions that (can) shape care relationships. Appropriate care is, thus, embedded in a web of formal and informal care relationships (see figure 1)—to HCPs, families, caregivers, spouses, insurance companies, other bureaucratic institutions, to oneself and one’s body, medication, aid devices, the physical environment, information, etc. Patients need to shape these relationships to receive the best possible care. The more successful this shaping, the more appropriate their care will be. Conversely, appropriate care enables relationship shaping, as patients are more able to engage in it if they are in a better physical or mental condition.

**Figure 2:**
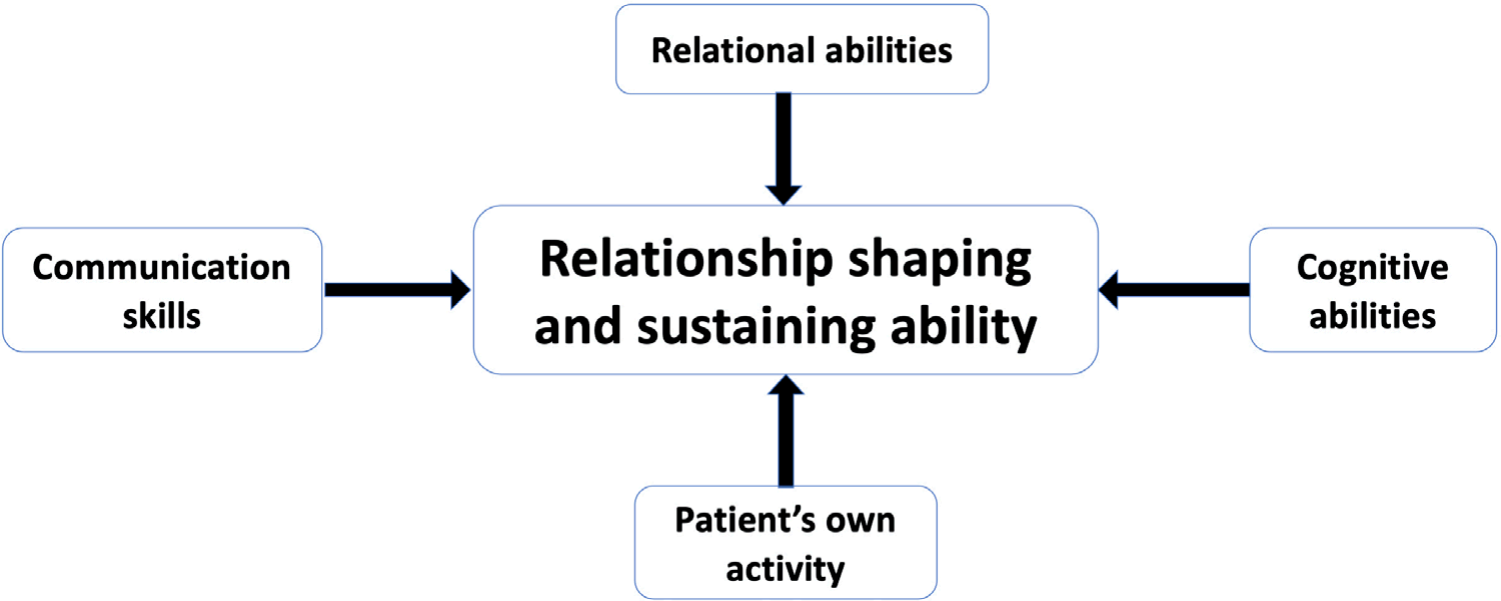
Components of a patient’s relationship shaping and sustaining ability.

Relationship shaping changes after a stroke according to the new situation and new needs. It also changes over the course of the illness. Only adaptable relationship shaping leads to continued appropriate care. The ability to shape relationships is a composite of several skills, such as engaging in and sustaining relationships, the ability to find and use information and communication skills (see figure 2). Patients’ own activity is central, for example, initiating relationships or obtaining care-improving information, as the following quotes illustrate:

“Although I also read a lot by myself, I ordered brochures, [down]loaded things online.” (f, late 50s, 3 years since stroke)

“I have […] some gadgets here, with which I exercise my hand every day [exercise balls etc. on kitchen table] […] yes, on days I don’t have physical therapy, I do something at home […]. Every day.” (f, mid 70s, 2.5 years since stroke)

Partners or caregivers can support this; HCPs (nurses, therapists, physicians, pharmacists, professional carers, etc.) usually only provide this support in specific situations, not systematically. However, they can provide crucial assistance, especially to those with poor relationship-shaping skills or those whose limitations due to stroke or other illnesses impede relationship shaping. The better HCPs are at relationship shaping, the better they will be able to discover patients’ unmet needs.

Healthcare structures can help or hinder the shaping and sustaining of relationships. This will occur more smoothly in a healthcare setting where HCPs have enough time, where there is continuity of care, where care is well organised so that patients do not need to use their relationship-shaping capacities for basic organisation and where connections to other HCPs are easily established. Conversely, relationship shaping is less likely to be successful if HCPs are pressed for time, the care relationship must be built anew in every consultation or the healthcare system makes it difficult to even establish a relationship, for instance, when physicians have reached full capacity. The following quote illustrates how healthcare structures can hinder relationship shaping. It shows a patient choosing not to receive care he is entitled to because of the way it is organised, thus forgoing care relationships:

Wife: “We wanted to do a nursing day care, once a week. (.) but for that, he needs two services: one to carry him downstairs [from the fifth floor, no elevator] and one to drive him there. […] The special transportation service may NOT drive him to day care. They would carry him downstairs, then he’d have to wait for the next service. In one of the nursing homes, she told me […] ‘He might have to […] wait for an hour downstairs.’ Obviously that put him off [the idea].” (m, early 70s, 4.5 years since stroke)

Conversely, the following quote illustrates how a well-organised healthcare system can enable relationship shaping:

“Well HERE in [village] we’re very well equipped with the outpatient clinic, the hospital. (.) Because at the hospital, there are consultation hours every day and on weekends it’s always possible to speak to someone RIGHT AWAY. I think that’s really great.” (f, mid 70s, 2.5 years since stroke)

Relationship shaping can beget new relationships, leading to a virtuous cycle of appropriate care, for example, when HCPs recommend other therapies or other HCPs. Conversely, not being able to shape relationships, for instance, when mobility issues prevent patients from leaving their apartments, can lead to a vicious cycle of inappropriate care. Instances of both phenomena are shown in the following quotes:

“About a year ago, I had [my physician] prescribe me an EMG-triggered stimulation device […] for the wrist and arm […] that my occupational therapist recommended.” (m, late 50s, 20 years since stroke)

Wife: “And now we had it with his teeth. He didn’t go to the dentist for four years, because he couldn’t get there.” (m, early 70s, 4.5 years since stroke)

### Strategies for shaping relationships

Study participants use multiple strategies to shape relationships. They also integrate earlier experiences into their relationship shaping, such as lessons from being caregivers themselves. They shape relationships on multiple tracks simultaneously, for instance, being on the waiting list for a care home while also maintaining good relationships with friends and neighbours who support them in daily life. They use information as a tool for relationship shaping that—ideally—tells them which relationships they need and how to best shape them. Persistence is another important strategy; for example, doing regular exercises to improve mobility can result in better mobility and a better relationship with their body, while persistence in appealing bureaucratic decisions regarding disability or caregiver support can lead to more access and services regarding disability support. Some participants shape relationships to their formal HCPs via their spouse, for example, by having them make appointments or find physicians for them.

An unexpected strategy was sparing relationships. Here, participants try to use personal relationships for care only when absolutely necessary, in order to preserve the social aspects of the relationship and ensure long-term support. An important consideration is not wanting to bother others, such as by asking for a ride to cultural or social activities. This strategy is also applied to formal care relationships, including only contacting HCPs when patients deem it truly necessary, with the accompanying danger of delaying (preventative) care. This strategy can have the desired effect of preserving relationships for future needs. However, it can also lead to social withdrawal and avoidance with no future benefit. This strategy is illustrated in the following quote:

“Well, one would LOVE to not become a burden to anyone. (.) And that’s why our lives are pretty secluded. […] I don’t want to ask for a ride every time I want to go somewhere. […] I don’t like asking […] because then I think: And now you’re a burden again.” (m, early 80s, 18 years since stroke)

### Factors and barriers influencing relationship shaping

Factors influencing a person’s ability to shape relationships relate to the individual as much as to others, to the relationships themselves and to the systems or institutions in which the relationship (shaping) takes place. These factors are listed in table 2.

**Table 2:** Factors influencing relationship shaping and sustaining

Factor category	Factors	Example
Factors related to patients	Supporting factors: Patients become/are good relationship partners They recognise the urgency of their care Special case: very low expectations → consider any care adequate and are rarely disappointed Inhibiting factors: Not realising that they are entitled to care will lead to less relationship shaping and less adequate care Lack of assertiveness can lead to care they do not consider adequate (more or less care than they want) Waning ability to shape relationships, such as due to illness and frailty	“We acted incorrectly with the diabetologist. Normally, there is a blood draw one week before the appointment […] so they have all the numbers when they see us. We often went to our appointment, but not the blood draw (.) UNTIL someone told us (.) ‘Get your act together and stick to our procedures!’ Completely understandable, […] since then that hasn’t happened anymore and […] if YOU […] asked Dr. [diabetologist] or Dr. [GP] about us, I think they’d tell you that we’re sick, but […] patients who don’t cause problems, who adhere to the rules.” (m, early 60s, 16 years since stroke)
Factors related to the other person or institution in a relationship	Supporting factors: Competence of the person opposite (professionally, regarding care, but also in relationship shaping) Reliability Inhibiting factors: Lack of partners with whom to shape relationships	“Especially with [GP], I feel VERY well cared for, a very competent physician, who thinks of everything, and that’s obviously important to me, to always be in good hands.” (m, late 50s, 20 years since stroke)
Factors related to the relationship itself	Supporting factors: Closeness of relationship Pre-existing relationship prestroke Inhibiting factors: No relationship shaping Changing relationship partners Communication deficit Relevant disagreements about care (can lead to inadequate care or failure of relationship)	“I had a GP, but she wouldn’t VACCINATE me (.) and since I’m on immunosuppressants (I: yes) I need the vaccines. So I looked for a physician who vaccinates […]I: And your old physician did not want to vaccinate you?B: No, she’s an anti-vaxxer.” (m, mid 60s, 13 years since stroke, transplant recipient)
Factors related to the system or institution in which the relationship takes place	Supporting factors: Sufficient time Steady flow of information Well-organised system/institution Inhibiting factors: Lack of time Slow/insufficient flow of information Disorganisation	“Especially bad? That was at an orthopedist’s, where I had to wait for four hours. (…) I told him ‘You know, Doctor, if you see six patients an hour, then you have ten minutes per patient.’ [He said] ‘Oh, but I can see 14 patients in an hour!’ (…) And then I was fed up.” (m, early 80s, 18 years since stroke)

Barriers to relationship shaping can be physical, financial, bureaucratic, psychological/emotional and missing information. Physical barriers include mobility issues and a lack of accessible infrastructure (eg, elevators), which lead to participants choosing to stay home, avoid social outings and postpone less urgent medical care, such as dental checkups, until this creates new medical issues. Copayments exceeding a patient’s budget or insurance not covering treatment that would facilitate relationship shaping, such as high-tech mobility devices, are financial barriers. Dealing with bureaucracy is a barrier when patients do not understand the rules of the bureaucratic system or deliberately do not follow them, which impedes relationship shaping with institutions. Psychological and emotional barriers, such as fear, depression or loneliness, also hamper relationship shaping. Lack of information hinders relationship shaping, as patients do not know that there are relationships they could shape to receive better care or because they do not know that they may be entitled to better care. The following quote illustrates several barriers at once:

“The problem was actually my health insurance. I’m insured privately […] they didn’t reimburse speech therapy at all (…) But back then, I hadn’t recovered enough, psychologically, to file an objection.” (f, late 50s, 3 years since stroke)

### Types

We found four types of relationship shaping and sustaining: successful, failed, irregular and selective. Definitions and characteristics are listed in table 3.

**Table 3:** Definitions and characteristics of relationship shaping and sustaining types

Type	Successful relationship shaping/sustaining	Failed relationship shaping/sustaining	Irregular relationship shaping/sustaining	Selective relationship shaping/sustaining
Definition	Relationships lead to adequate care. A patient’s individual concerns and requests shape how care is provided.	Relationships do not lead to adequate care. Patients’ individual situation and concerns are largely disregarded.	Intermediate between successful and failed. Relationships could be better, but care is somewhat adequate.	Patients select areas to pursue relationship shaping or select relationships to shape (extreme case: assisted dying).
Signs and symptoms of this type	Patients feel that they are ‘in good hands’ Patients are empowered to make their own decisions HCPs do more than necessary Patients can access support and care without problems when it is needed	Patients feel left alone with their health issues Patients feel that physicians are ‘tinkering’ with their health No chemistry Receive unwanted care Care appears pointless	Solutions are not very good Provision more reliable if patients circumvent healthcare system Tried many things, none work Care not completely adequate	Patients make a conscious choice, such as no longer participating in cancer screening or adhering to exercise and nutrition recommendations but continuing to smoke
Cause/ reasons	Patients recognise the urgency for participating in their healthcare They become good partners Special case: very low expectations (so low that any care is considered adequate) Relationship partners are competent Reliable HCPs	Communication deficit No partners for relationships available Incompetent partners Low assertiveness No efforts to shape the relationship (on either side) Disagreements relevant to care Patients do not realise that they are entitled to care	Changing partners Disorganisation Lack of time Deteriorating ability to shape relationships Differing expectations on both sides Lack of information	Involuntary selection (eg, because few potential partners are left) Patients do their own thing (ie, gym instead of physical therapy) Patients want to manage on their own
Conducive situations	Long-term relationships Time: interactions are not rushed	Emergencies/acute care Lack of time	New or disorganised settings Complex needs	Empowered, informed patients
Example	“With [GP] I feel VERY, VERY well cared for […] she STILL wants to see me […] every three months, […] I really feel very looked after.” (f, late 50s, 3 years since stroke)	“Regarding the stroke, I wish that you (…) YES that you’re not […] so alone (…) and then you think, how do I find a neurologist, how do I go on, where do I find help? Because HELP! I’m (.) unwell so to speak.” (f, mid 70s, 2 years since stroke)	“[The home care nurse] sometimes already comes shortly after six a.m. and then I get up and they dress me. That’s why I did this [points to her couch with a blanket and pillow], and sometimes I lie down here and then I sometimes sleep until 10.45 a.m.” (f, early 90s, 6.5 years since stroke)	“After the stroke I said, gynecological checkups are no longer relevant for me. […] Sometimes my husband says: ‘Hey, we must go to the doctor, get to the bottom of this.’ (…) THAT is superfluous. I [am] so busy with the stroke sequelae that I neglect everything else, as it were.” (f, mid 70s, 2.5 years since stroke)

HCP, healthcare professional.

## Discussion and conclusion

### Discussion

From our interviewees’ perspectives, receiving appropriate care ultimately relates to how relationships are shaped and sustained. We found that patients’ ability to shape relationships may be essential for receiving appropriate long-term care after stroke since their own activities are central for shaping care relationships. This process can be helped or hindered by healthcare system organisation. Patients use different strategies to shape relationships; multiple factors and barriers contribute to their success or lack thereof. This leads to successful, failed, irregular or selective relationship shaping. Relationship shaping may be indispensable for both formal and informal care relationships, as appropriate care consists of both. Thus, relationship shaping and sustaining is not ‘nice to have’ or a matter of making interactions more pleasant, but—as our analysis demonstrates—may be in fact vital for appropriate care. We, therefore, assume that the quality of relationships and their shaping and sustaining can determine the quality of care, which, in turn, could influence a patient’s biopsychosocial situation—their mobility, communication skills, likelihood of stroke recurrence, etc.

The initial thematic analysis on ‘unmet needs’ revealed the complexity of how patients talk about care. Rather than improving our understanding of patients’ struggles, doings and experiences when talking about their care, however, analysis from the perspective of ‘unmet needs’ suggests a receptive—somewhat passive—patient who articulates a need that *should* be addressed by a healthcare system, but which is not being taken care of by this system. The term ‘unmet needs’ is defined externally from a system perspective. However, it misconstrues what long-term care is actually *about* in terms of patients’ experience and how it works in practice. A focus on ‘unmet needs’ leads to a kind of to-do list for HCPs: optimise pharmacotherapy, start physical therapy, contact a social worker, etc. Activity and passivity in the patient–provider relationship can have reciprocal effects, with passive healthcare services leading to more passive patients and active services empowering patients to more self-management.^27^ Active relationship shaping may be one of the mechanisms explaining this. An approach that looks at ‘shaping relationships’ is more open and, thus, leads to more answers to the question of how to improve long-term care. It centres patients’ active and crucial role in the process of long-term care by viewing ‘shaping relationships’ as a foundation for active patient participation and better reflects their experience of it. Compared with ‘unmet needs’, this approach takes a step back to better understand how long-term care works in practice and how to improve it.

While the importance of relationships is recognised elsewhere, studies examining care relationships usually consider the benefits of a good relationship or specific relationships in specific settings (eg,^28^,^33^), rather than the importance of the ability to shape relationships for overall care. Care takes place over time and in a web of relationships between the patient and formal and informal care providers, and among providers themselves (see figure 1). Active relationship shaping expands the web, strengthens and untangles its strands, cuts unhelpful ties, etc. A larger, denser web leads to more care, more options, more fallback plans and more support. For appropriate care, the web should have the strength and elasticity to catch patients in crisis, as well as the durability to function in daily life.

Patient-centred care^19 34 35^ implicitly recognises the importance of relationships, but does not focus on patients’ abilities (or lack thereof) to influence them in order to improve their care. This is a missing link that could explain why current models of patient-centred care work better for some patients than for others: those with good relationship-shaping abilities benefit directly from patient centredness, while those with poorer relationship-shaping abilities need specific support in this area.

Research on the care relationship often focuses on how physicians’ behaviour and strategies contribute to good relationships and relationship shaping, such as valuing individuals, collaborating with and empowering them or offering emotional support.^28^ In a review of interpersonal patient–provider interventions, 67% of interventions targeted providers.^15^ A similar focus on how HCPs communicate is also common in communication training in medical schools and beyond. However, our interviews show that, for long-term care, patients’ behaviour (or that of their proxies) in shaping relationships may be more important. The ability to shape relationships is a composite of relational abilities, communication skills, cognitive abilities and a patient’s own activity (see figure 2). Patients are ‘active agents’ in managing their relationship with their GP^36^; when these care relationships lead to appropriate care, an important factor is the active role that patients or their proxies play in shaping them. This can be extrapolated to other care relationships.

Patients’ experience of continuity of care may be influenced by relationship shaping even without relational continuity, that is, seeing the same HCPs multiple times. Knowledge, attitudes and experience of HCPs as well as their knowledge of local services and their willingness to take on a patient’s problem and to go beyond the requirements of their role were found to be important for patients’ experience of management continuity in one-off encounters.^37^ We hypothesise that these factors will vary depending on relationship shaping.

We need to understand how care relationships work, how they can be shaped, their commonalities, differences and interactions and how they constitute a web of care relationships—because appropriate care is more complex than the sum of individual care relationships. The current iteration of long-term care after stroke, however, relies on patients’ relationship-shaping abilities while being unaware of the importance of this skill. If we wish to improve long-term care, this is a promising point of departure.

Our findings suggest a two-pronged approach: supporting patients in shaping relationships and organising care systems that do not rely on this ability. Our study calls for a shift in perspective among HCPs: they need to recognise the importance of relationship shaping *by patients themselves*, and it needs to become a part of healthcare curricula. Only then can HCPs successfully support patients in this process and use their own relationships to improve care. This is especially important for patients whose natural relationship-shaping abilities are less developed or for those whose illness impairs relationship shaping. We need to invest in patients’ relationship-shaping abilities by encouraging their attempts, making the concept part of informational material and providing care that improves the skills that constitute the ability to shape relationships. This would be aided by enhancing HCPs’ relationship-shaping skills, especially at care intersections. This could better attune HCPs to patients’ needs and especially to patients who cannot voice their needs because of poor relationship-shaping skills.

One reason for the importance of relationship shaping may be structural problems in healthcare systems. There are institutional barriers to care, such as lack of information, insurance and bureaucracy, and patients with good relationship-shaping abilities may be able to navigate around these barriers more easily than others. The aim of our research is not to hold patients accountable for lacking this ability or to assign them additional responsibility for their care. It is to highlight the importance of relationship shaping for long-term care within the context of the German healthcare system. Policymakers and HCPs need to understand how crucial this ability is for adequate long-term care after stroke so that reforms can be implemented to make care less dependent on patients’ social skills in the future.

### Strengths and limitations

A strength of this study is its focus on the patient perspective, as patients have the best overview of long-term care. A wide range of time since the last stroke (2–20 years) gave us a broad overview of secondary prevention and long-term care. Recruitment through trusted doctors and therapists simplified inclusion. An open, qualitative approach enabled a wide range of findings with regard to care relationships and relationship shaping. We reached theoretical saturation in our analysis. Grounded theory allowed us to illuminate complex processes. One person conducted all interviews, which ensured the continuity of data collection.

A limitation of the study is related to recruitment in healthcare settings, meaning that we did not include patients without GP or PT care. It is also likely that recruiters only approached patients with whom they are comfortable, potentially leading to a selection bias. Thus, our sample may be biased towards participants who are good at shaping relationships and/or have better access to care. This may limit generalisability—participants approached through other settings, for example, a rural area as opposed to a large metropolitan area, may have told different stories and other core categories could have emerged. The skill of forging relationships may also be relevant during interviews; further studies should consider other methods. We only recruited participants that are able to speak and can, therefore, only ascertain how the verbally competent shape care relationships. 15 interviews were conducted, meaning that the results could have been different with a larger sample. However, limiting the number of interviews allowed for very thorough analysis and we reached theoretical saturation. We focused on long-term care after stroke, but it is possible that relationship shaping is generally applicable to long-term care for chronic illnesses. Other factors influence the experience of long-term care, such as institutional barriers or the allocation of resources, but this was beyond the scope of this article.

### Conclusion

Shaping and sustaining relationships may be essential in long-term care after stroke. This composite skill includes engaging in and sustaining relationships, finding and using information and communication. There is a need for innovative models of care that put the onus of relationship shaping on others. Healthcare systems need to recognise that relationship shaping is crucial for adequate long-term care and that creating a relationship-friendly environment will in turn improve care.

## Supplementary material

10.1136/bmjopen-2024-090206 online supplemental file 1

## Data Availability

Data are not publicly available.
