# Supplementary material for: How do patients access and experience long-term care after stroke in the German healthcare system? A qualitative interview study
Source: BMJ Open. 2025 Jun 30;15(6):e090206. doi: 10.1136/bmjopen-2024-090206 (PMC12211827; doi:10.1136/bmjopen-2024-090206)
Supplement: online supplemental file 1 [file bmjopen-15-6-s001.pdf]

How do patients access and experience long-term care after stroke? A qualitative interview study – Supplementary files

Banzhoff et al.

Supplementary table 1: Summary of interview questions

Summary of interview questions
• What has changed for you after your stroke?<br>• What activities can you no longer do/participate in that used to be easy?<br>• Do you have any other illnesses, and if so, which?<br>• Do you feel well informed about your conditions? Why (not)?<br>• Who supports you?<br>• Do you have health-related goals / still hope for improvements? How can you influence this?<br>• How self-sufficient are you? Please describe problems you have in daily life.<br>• Did you receive disability support after your stroke and were there problems in accessing it?<br>• Which physicians do you see and why? Would you like to see them more often? Who coordinates care when you are treated by multiple physicians?<br>• How long have you been a patient with your GP?<br>• Was there a time since the stroke when you weren't in treatment at all?<br>• What is important to you regarding medical care? Has this changed since the stroke?<br>• Have you recently had physical, occupational, or speech therapy? If not, why not?<br>• Who do you contact when you are not feeling well?<br>• Who cares for you apart from physicians?<br>• Which other care institutions do you know about and why do/don't you use them?<br>• What should a person supporting you be able to do?<br>• If you could have additional support, what would you ask for?<br>• What do you wish for regarding your health / regarding your stroke?<br>• Are your opinions and wishes regarding care taken into account?<br>• What would need to happen for you to feel well cared for?
