# Supplementary material for: How do patients access and experience long-term care after stroke in the German healthcare system? A qualitative interview study
Source: BMJ Open. 2025 Jun 30;15(6):e090206. doi: 10.1136/bmjopen-2024-090206 (PMC12211827; doi:10.1136/bmjopen-2024-090206)
Supplement: Supplementary file 2 [file bmjopen-2024-090206.reviewer_comments.pdf]

PEER REVIEW HISTORY

BMJ Open publishes all reviews undertaken for accepted manuscripts. Reviewers are asked to complete a checklist review form and are provided with free text boxes to elaborate on their assessment. These free text comments are reproduced below.

ARTICLE DETAILS

Title (Provisional)

How do patients access and experience long-term care after stroke in the German healthcare system? A qualitative interview study

Authors

Banzhoff, Sofia; Heintze, Christoph; Doepfmer, S; Holmberg, Christine

---

VERSION 1 - REVIEW

**Reviewer**	**1**
**Name**	**Mant, Jonathan**
**Affiliation**	**University of Cambridge, General Practice and Primary Care Research Unit**
**Date**	**13-Aug-2024**
**COI**	**None**

I enjoyed reading this qualitative study that highlights a neglected area in stroke research – longer term relationship of stroke survivors with care – and identifies an issue – referred to here as 'shaping relationships' – as key. Again, this issue has not been widely covered in the literature. I thought it was well written with sparing use of quotations and a couple of helpful summary tables.

While not a qualitative researcher, it seemed methodologically sound to me, and to present important findings that are of general interest.

I have a few minor comments:

-In abstract, it is not clear who 'shaping relationships' applies to: patient and health care professional; patient and family/friends.

Introduction:

-p2 presumably 12.2m and 101m refers to global population?

-p2: how is 2 year risk of recurrence (16.1%) higher than 10 year risk of recurrence?!!!

-p3: 'Care can also improve quality of life….' This statement needs a reference.

Methods:

-Justification for the target number of participants (15) seems rather weak.

Results:

-It is not always clear to me how the quotes illustrate the declared finding. At the top of p10, it refers to patients initiating relationships, but the quotes do not relate to this. At the bottom of p10, there is a comment about building relationships and how the health care system can interfere with this. The illustrative quote on p11 certainly describes limitations of provided care, but it is not explicit to me what this has to do with relationship forming.

Discussion:

-Well argued. There is some resonance with the findings of Pindus et al PLOS one (2018) with regard to active and passive participation.

Reviewer 2

Name Kinoshita, Shoji

Affiliation The Jikei University School of Medicine, Department of Rehabilitation Medicine

Date 30-Oct-2024

COI no COI

Major concerns

The approach of this paper focuses on the patients' ability to actively shape relationships. However, this perspective implies that if patients lack this ability or are unable to utilize it, accessing care becomes difficult, which raises concerns. This viewpoint risks placing part of the responsibility for inadequate care on the patients, which should be carefully considered.

In reality, access to long-term care depends not only on the individual patient's capabilities but also heavily on structural issues within social systems and healthcare infrastructure. For example, if sufficient information is not provided, if the procedures to access care are complex, or if the system itself is difficult to navigate, even patients who try to build relationships may not receive adequate support.

If the role of social systems is underestimated and the quality of care is overly dependent on patients' efforts and abilities, there is a risk of creating inequalities. Therefore, improvements in the overall healthcare system are necessary. It is crucial to question how well-designed social welfare and healthcare systems are, particularly in terms of accessibility for older adults and people with disabilities. Ideally, systems should be designed to support patients and facilitate relationship-building. In this regard, I believe the study should delve deeper into the responsibilities of healthcare providers and the system itself.

The study briefly touches on healthcare system challenges but doesn't delve deeply enough into the institutional barriers (e.g., insurance issues, bureaucracy) that might impede relationship shaping. Expanding on this could strengthen the discussion.

An explanation of the German care system would be highly beneficial for readers outside of Germany. Since healthcare and long-term care systems differ significantly from country to country, providing a detailed explanation of the German context would help readers better understand the study's findings and clinical implications.

By explaining Germany's long-term care insurance system, the roles of care advisors, home care services, and primary care physicians, readers would be able to make comparisons with their own systems. This would allow them to reflect on how the findings may apply within their own countries and consider possible improvements based on Germany's successes or challenges.

Providing this kind of background information would enhance the international relevance of the research and offer valuable insights for countries with different social systems. This could also facilitate broader discussions on how the findings might be adapted to other healthcare settings.

The recruitment of participants through general practitioners (GPs) and physical therapists (PTs) with pre-existing relationships might introduce selection bias, as it could skew results toward those patients with better access to care or stronger relationships with health providers . This limits the generalizability of the findings to the broader stroke population who may have different care experiences or less support.

The study's reliance on a small sample size (n=15) with participants recruited from a specific region (Berlin) might limit the external validity. Including a more diverse population, such as patients from rural or less resourced areas, would provide a more comprehensive understanding of long-term stroke care

Minor concerns

The analysis process, particularly in relation to the grounded theory approach, would benefit from further elaboration. A more transparent discussion of how the open and focused coding led to the development of the "relationship shaping" concept would enhance the study's methodological rigor

Although the discussion mentions that "a broad range of perspectives and insights were gained," the actual content seems to focus almost exclusively on the formation of relationships, with little discussion of other aspects. While it is natural for the discussion to concentrate on the primary theme of relationship-building, the claim of "broad perspectives" might feel somewhat misleading.

For example, other important factors such as institutional barriers, resource allocation, or quality metrics in care could also play a significant role in the long-term care experience, but these are not addressed in the discussion. As a result, the discussion is limited to the topic of

relationship-building, leaving out a more comprehensive view of the long-term care process.

**Reviewer**	**3**
**Name**	**Visvanathan, Akila**
**Affiliation**	**The University of Edinburgh**
**Date**	**26-Mar-2025**
**COI**	**Not applicable**

appears appropriate; it is difficult to make concrete conclusions from a small qualitative study and if data saturation was truly achieved - worth reflecting on this further and if other qualitative analysis may have been more appropriate e.g. thematic analysis with two reviewers reviewing the manuscripts.

VERSION 1 - AUTHOR RESPONSE

Reviewer: 1
Prof. Jonathan Mant, University of Cambridge
Comments to the Author:
I enjoyed reading this qualitative study that highlights a neglected area in stroke research – longer term relationship of stroke survivors with care – and identifies an issue – referred to here as 'shaping relationships' – as key. Again, this issue has not been widely covered in the literature. I thought it was well written with sparing use of quotations and a couple of helpful summary tables.
While not a qualitative researcher, it seemed methodologically sound to me, and to present important findings that are of general interest.
RESPONSE: Thank you very much for your valuable comments and your positive feedback on our manuscript!
I have a few minor comments:
-In abstract, it is not clear who 'shaping relationships' applies to: patient and health care professional; patient and family/friends.

RESPONSE: Thank you for your comments and suggestions. We've changed the wording in the results section of the abstract to make this clearer. It now reads:
"'Shaping relationships' was the core category extracted from the data as a necessary component to receive appropriate long-term care after stroke. Care is embedded in relationships that must be managed primarily by the study participants and their proxies in the German setting."
Introduction:
-p2 presumably 12.2m and 101m refers to global population?
RESPONSE: Thank you for pointing this out. We've changed the wording to make this clear: It now reads (p. 2):
"The prevalence and incidence of stroke remain high despite prevention efforts. In 2019 the incidence was estimated to be 12.2 million strokes worldwide and prevalence was assumed

to consist of 101 million persons worldwide. If these trends continue the estimated global prevalence rate for stroke by 2050 is 200 million persons [1]."

-p2: how is 2 year risk of recurrence (16.1%) higher than 10 year risk of recurrence?!!!

RESPONSE: We've double-checked the numbers and they are cited correctly. The systematic review and meta-analysis cited here (Lin et al, 2021, Neurological sciences) notes substantial heterogeneity between studies (except at 10 years). The recurrence rates at 2 and 10 years are calculated based on different studies and data sets. The authors attribute the heterogeneity to differences in data stratified by age (with lower recurrence rates in patients younger than 50), variations in classification criteria (stroke type), differences in follow-up methods and variation in research settings. The recurrence risk at 12 years is 39.7%. In order to make this less confusing to the reader, we have changed this accordingly. The passage now reads (p. 2):

"Furthermore, the risk of stroke recurrence is 16.1% two years after the initial stroke, 14.8% after five years and 39.7% after twelve years [3]."

-p3: 'Care can also improve quality of life….' This statement needs a reference.

RESPONSE: Thank you for bringing this to our attention. We've added a reference. The passage now reads:

Care and rehabilitation can also improve quality of life (QoL) [6]. (p. 3)

Methods:

-Justification for the target number of participants (15) seems rather weak.

RESPONSE: We've expanded the justification. The passage now reads:

"Based on a review [24] one may assume that the most crucial information in qualitative interview studies is gained within the first six interviews, with the rate of new information declining sharply following an asymptotic curve. Our aim was to interview 15 participants to get an overview of different long-term care situations after stroke and ensure data saturation for our research questions. Previous research by the authors SD and CH also supports such sample sizes [25]." (p. 7)

Results:

-It is not always clear to me how the quotes illustrate the declared finding. At the top of p10, it refers to patients initiating relationships, but the quotes do not relate to this. At the bottom of p10, there is a comment about building relationships and how the health care system can interfere with this. The illustrative quote on p11 certainly describes limitations of provided care, but it is not explicit to me what this has to do with relationship forming.

RESPONSE: The quotes at the top of p. 10 are meant to illustrate the centrality of patients' own activity, which we've now made explicit in the text. The passage now reads (p.10):

"Patients' own activity is central; for example, initiating relationships or obtaining care-improving information, as the following quotes illustrate:

"Although I also read a lot by myself, I ordered brochures, [down]loaded things online." (f, 57, 3 years since stroke)

"I have […] some gadgets here, with which I exercise my hand every day [exercise balls etc. on kitchen table] […] yes, on days I don't have physical therapy, I do something at home […]. Every day." (f, 74, 2.5 years since stroke)"

The quote on p. 11 is meant to illustrate how structures in the healthcare system hinder relationship shaping. It shows a situation where a patient chooses not to receive care he is entitled to because of the way it is organized. He thus forgoes the relationship the nursing

day care could offer – opting out of relationship shaping for logistical reasons. The passage now reads (p. 11):
“The following quote illustrates how health care structures can hinder relationship shaping. It shows a patient choosing not to receive care he is entitled to because of the way it is organized, thus forgoing care relationships:
Wife: “We wanted to do a nursing day care, once a week. (.) but for that, he needs two services: one to carry him downstairs [from the fifth floor, no elevator] and one to drive him there. […] The special transportation service may NOT drive him to day care. They would carry him downstairs, then he’d have to wait for the next service. In one of the nursing homes, she told me […] ‘He might have to […] wait for an hour downstairs.’ Obviously that put him off [the idea].” (m, 73, 4.5 years since stroke)
Conversely, the following quote illustrates how a well-organized healthcare system can enable relationship shaping:
“Well HERE in [village] we’re very well equipped with the outpatient clinic, the hospital. (.) Because at the hospital, there are consultation hours every day and on weekends it’s always possible to speak to someone RIGHT AWAY. I think that’s really great.” (f, 74, 2.5 years since stroke)”
We’ve also double-checked all other in-text quotes and made explicit what they illustrate (pages 12, 13 and 15).
Discussion:
-Well argued. There is some resonance with the findings of Pindus et al PLOS one (2018) with regard to active and passive participation.
RESPONSE: Thank you for your comment and suggestion. We have added this to the discussion (p. 18). The passage reads:
“The term ‘unmet needs’ is defined externally, from a system perspective. However, it misconstrues what long-term care is actually *about* in terms of patients’ experience and how it works in practice. A focus on ‘unmet needs’ leads to a kind of to do list for HCPs: optimize pharmacotherapy, start physical therapy, contact a social worker, etc. Activity and passivity in the patient-provider relationship can have reciprocal effects, with passive healthcare services leading to more passive patients and active services empowering patients to more self-management [27]. Active relationship shaping may be one of the mechanisms explaining this. An approach that looks at ‘shaping relationships’ is more open and thus leads to more answers to the question of how to improve long-term care. It centers patients’ active and crucial role in the process of long-term care by viewing ‘shaping relationships’ as a foundation for active patient participation and better reflects their experience of it.”

Reviewer: 2
Dr. Shoji Kinoshita, The Jikei University School of Medicine
Comments to the Author:
Major concerns
# The approach of this paper focuses on the patients’ ability to actively shape relationships. However, this perspective implies that if patients lack this ability or are unable to utilize it, accessing care becomes difficult, which raises concerns. This viewpoint risks placing part of the responsibility for inadequate care on the patients, which should be carefully considered.

In reality, access to long-term care depends not only on the individual patient's capabilities but also heavily on structural issues within social systems and healthcare infrastructure. For example, if sufficient information is not provided, if the procedures to access care are complex, or if the system itself is difficult to navigate, even patients who try to build relationships may not receive adequate support.
If the role of social systems is underestimated and the quality of care is overly dependent on patients' efforts and abilities, there is a risk of creating inequalities. Therefore, improvements in the overall healthcare system are necessary. It is crucial to question how well-designed social welfare and healthcare systems are, particularly in terms of accessibility for older adults and people with disabilities. Ideally, systems should be designed to support patients and facilitate relationship-building. In this regard, I believe the study should delve deeper into the responsibilities of healthcare providers and the system itself.
The study briefly touches on healthcare system challenges but doesn't delve deeply enough into the institutional barriers (e.g., insurance issues, bureaucracy) that might impede relationship shaping. Expanding on this could strengthen the discussion.
RESPONSE: Thank you very much for your valuable comments and your positive feedback on our manuscript!

We've added a passage addressing this concern to the discussion on p. 20-21. It reads:
"One reason for the importance of relationship shaping may be structural problems in healthcare systems. There are institutional barriers to care, such as lack of information, insurance, bureaucracy, and patients with good relationship-shaping abilities may be able to navigate around these barriers more easily than others. The aim of our research is not to hold patients accountable for lacking this ability or to assign them additional responsibility for their care. It is to highlight the importance of relationship shaping for long-term care within the context of the German healthcare system. Policy makers and HCP need to understand how crucial this ability is for adequate long-term care after stroke, so that reforms can be implemented to make care is less dependent on patients' social skills in the future."

# An explanation of the German care system would be highly beneficial for readers outside of Germany. Since healthcare and long-term care systems differ significantly from country to country, providing a detailed explanation of the German context would help readers better understand the study's findings and clinical implications.
By explaining Germany's long-term care insurance system, the roles of care advisors, home care services, and primary care physicians, readers would be able to make comparisons with their own systems. This would allow them to reflect on how the findings may apply within their own countries and consider possible improvements based on Germany's successes or challenges.
Providing this kind of background information would enhance the international relevance of the research and offer valuable insights for countries with different social systems. This could also facilitate broader discussions on how the findings might be adapted to other healthcare settings.
RESPONSE: Thank you for this suggestion! We've added an explanation to the introduction on p. 3. The passage reads:
"Most acute stroke patients in Germany are treated by neurologists in specialized stroke units in hospitals and many patients attend stroke-specific in- or outpatient rehabilitation

after that [8]. There are no structured aftercare programs following rehabilitation; GPs tend to be the main HCP for follow-up care. Problems can arise as the German healthcare system has a more rigid separation into sectors than other countries. Physicians working in hospitals only see inpatients, whereas follow-up care takes place in ambulatory practices of GPs or other specialists. This can lead to logistical issues and gaps in care [8]. Social network size was found to be positively associated with QoL (quality of life) in the first year after discharge from rehabilitation [9]. Insurance is a lower barrier than in other countries as more than 90% of the population are insured via statutory health insurance and most others insured privately [10]."

# The recruitment of participants through general practitioners (GPs) and physical therapists (PTs) with pre-existing relationships might introduce selection bias, as it could skew results toward those patients with better access to care or stronger relationships with health providers. This limits the generalizability of the findings to the broader stroke population who may have different care experiences or less support.
RESPONSE: Thank you for pointing this out. We've discussed this on p. 21 and have made the passage clearer. It now reads:
"A limitation of the study is related to recruitment in health care settings, meaning we did not include patients without GP or PT care. It is also likely that recruiters only approached patients with whom they are comfortable, potentially leading to a selection bias. Thus our sample may be biased towards participants who are good at shaping relationships and/or have better access to care. This may limit generalizability – participants approached through other settings, for example a rural area as opposed to a large metropolitan area, may have told different stories and other core categories could have emerged."

# The study's reliance on a small sample size (n=15) with participants recruited from a specific region (Berlin) might limit the external validity. Including a more diverse population, such as patients from rural or less resourced areas, would provide a more comprehensive understanding of long-term stroke care
RESPONSE: We've discussed this on p. 21 + 22. The passages read:
"participants approached through other settings, for example a rural area as opposed to a large metropolitan area, may have told different stories and other core categories could have emerged." (p. 20)
"Fifteen interviews were conducted, meaning that the results could have been different with a larger sample." (p. 21)
We have also added a clarifying passage to the methods section on p. 7. It reads:
"Based on a review [24] one may assume that the most crucial information in qualitative interview studies is gained within the first six interviews, with the rate of new information declining sharply following an asymptotic curve. Our aim was to interview 15 participants to get an overview of different long-term care situations after stroke and ensure data saturation for our research questions. Previous research by the authors SD and CH also supports such sample sizes [25]." (p. 7)

Minor concerns
# The analysis process, particularly in relation to the grounded theory approach, would benefit from further elaboration. A more transparent discussion of how the open and

focused coding led to the development of the "relationship shaping" concept would enhance the study's methodological rigor
RESPONSE: Thank you for calling this to our attention. We've elaborated and specified the coding process to show how it led to the core category. The passage on p. 8-9 now reads:
"Throughout this process, the first and last author held data sessions at every step of the analysis process to discuss identified passages, coding, the coding system, memos and categorizations. They coded passages together and discussed the first author's codes of other passages in detail. Disagreements were resolved via discussions until they reached a consensus.
In the review of categories and subcategories and detailed discussion by the first and last author, a core category emerged that encompassed all categories and subcategories and explained the unique challenges of long-term care after stroke. This was tested by writing memos linking each category and subcategory to the core category. Preliminary results were discussed with several research groups and colloquiums at our institution."

# Although the discussion mentions that "a broad range of perspectives and insights were gained," the actual content seems to focus almost exclusively on the formation of relationships, with little discussion of other aspects. While it is natural for the discussion to concentrate on the primary theme of relationship-building, the claim of "broad perspectives" might feel somewhat misleading.
For example, other important factors such as institutional barriers, resource allocation, or quality metrics in care could also play a significant role in the long-term care experience, but these are not addressed in the discussion. As a result, the discussion is limited to the topic of relationship-building, leaving out a more comprehensive view of the long-term care process.
RESPONSE: While 'shaping relationships' emerged as the main theme, there were of course other aspects that were addressed as well, as you rightly point out. We have now made the focus clearer in the discussion. The passage now reads (p. 21):
"An open, qualitative approach enabled a wide range of findings with regard to care relationships and relationship shaping."
We've also discussed the point you made here as a limitation. The passage reads (p. 22):
"Other factors influence the experience of long-term care, such as institutional barriers or the allocation of resources, but this was beyond the scope of this manuscript."

Reviewer: 3
Dr. Akila Visvanathan, The University of Edinburgh
Comments to the Author:
appears appropriate; it is difficult to make concrete conclusions from a small qualitative study and if data saturation was truly achieved - worth reflecting on this further and if other qualitative analysis may have been more appropriate e.g. thematic analysis with two reviewers reviewing the manuscripts.
RESPONSE: Thank you very much for your valuable comments and your positive feedback on our manuscript!

We detail why data saturation was reached on page 9 and discuss it in the context of strengths and limitations on p. 22. The passage reads:

“The core category was developed further by applying it to the ten remaining interviews; this helped to identify its properties and patterns. The analysis of the last five interviews did not add any new aspects to the developed concept [26].” (p. 9)

We discuss why we deemed thematic analysis not suited to the data on pages 8 and 18. The passages read:

“Initially, only a thematic analysis regarding unmet needs in long-term care after stroke was envisioned. However, to adhere to the principle of openness and responsiveness to the data as important quality assurance criteria of qualitative research, we complemented this with a more open-ended and question-focused analysis related to a grounded theory approach once we saw the richness of the data with regards to our research questions and the limitations a sole thematic analysis would have posed. Thus, rather than focusing on a pre-conceived notion of ‘unmet needs,’ we investigated patients’ experiences of care. To be true to the data, the data were analyzed according to the following questions: What does it mean from patients’ perspectives to access and experience long-term care after stroke? How do they experience being cared for, receiving support for this care and receiving care? Following the grounded theory approach, the aim then became to develop a core category that explains the data in relation to care.” (p. 8)

“The initial thematic analysis on ‘unmet needs’ revealed the complexity of how patients talk about care. Rather than improving our understanding of patients’ struggles, doings and experiences when talking about their care, however, analysis from the perspective of ‘unmet needs’ suggests a receptive – somewhat passive – patient who articulates a need that *should* be addressed by a health care system, but which is not being taken care of by this system. The term ‘unmet needs’ is defined externally, from a system perspective. However, it misconstrues what long-term care is actually *about* in terms of patients’ experience and how it works in practice. A focus on ‘unmet needs’ leads to a kind of to do list for HCPs: optimize pharmacotherapy, start physical therapy, contact a social worker, etc. Activity and passivity in the patient-provider relationship can have reciprocal effects, with passive healthcare services leading to more passive patients and active services empowering patients to more self-management [27]. Active relationship shaping may be one of the mechanisms explaining this. An approach that looks at ‘shaping relationships’ is more open and thus leads to more answers to the question of how to improve long-term care. It centers patients’ active and crucial role in the process of long-term care by viewing ‘shaping relationships’ as a foundation for active patient participation and better reflects their experience of it. Compared to ‘unmet needs’ this approach takes a step back to better understand how long-term care works in practice and how to improve it.” (p. 18)

We’ve added further references to the literature to contextualize our findings on pages 18 and 20. The passages read:

“Activity and passivity in the patient-provider relationship can have reciprocal effects, with passive healthcare services leading to more passive patients and active services empowering patients to more self-management [27]. Active relationship shaping may be one of the mechanisms explaining this.” (p. 18)

“Patients’ experience of continuity of care may be influenced by relationship shaping even without relational continuity, i.e. seeing the same HCPs multiple times. Knowledge, attitudes and experience of HCPs as well as their knowledge of local services and their willingness to take on a patient’s problem and to go beyond the requirements of their role were found to be important for patients’ experience of management continuity in one-off

encounters [37]. We hypothesize that these factors will vary depending on relationship shaping." (p. 20)
